# Diseases of the Heart and Arteries, Their Causes, Nature, and Treatment

**Published:** 1895-11

**Authors:** 


### DISEASES OF THE HEART AND ARTERIES, THEIR CAUSES, NATURE, AND TREATMENT. By John H. Clarke, M. D., C. M., Edinburgh, Editor of *The Homœopathic World.* London: E. Gould & Son, 59 Moorgate Street, E. C. 1895.

In his preface the author says: "As my object is to deal more especially with the *therapeutics* of heart disease, I shall only devote to the consideration of the pathology of diseases of the heart and its vessels just so much space as is needed for my purpose." Accordingly, on perusing the book, we find that the author has given outline sketches of the various diseases and then followed these descriptions with the recital of cases, which were either cured outright or signally benefited by the homœopathic remedies. Much reliance is placed by the author upon the use of Iodide of Arsenic in the treatment.

The reader is not to infer from this statement that he regards Iodide of Arsenic as a specific in heart cases to the exclusion of all other remedies. On the contrary, he recites numerous cases where Iodide of Arsenic was not given at all, but other remedies administered, according to the indications. Many of his cases are illustrated by diagrams of the pulse taken with the sphygmograph.

He has taken up the reports made by the old school of the treatment of heart cases, by their new remedy Thyroidin, which is prepared from the thyroid gland of the sheep, and collecting the symptoms reported as produced by the poisonous effect of this drug in too large doses, he makes out quite a symptomatology that could be made very useful by homœopathic physicians in prescribing this medicine in potency. Dr. Clarke proposes to class this remedy as a "Sarcode," a title which seems very appropriate. He gives cases in which this "Sarcode" was very successful in the third decimal potency.

The author defines curative action by simply quoting and adopting Hahnemann's definition, and then with true Hahnemannian instinct declaring that tumors should be cured by Homœopathy. Differences of opinion concerning curative treatment are ingeniously explained, on page 140, as follows:

"Much of the disputing that has taken place over the proper method of selecting specifics might have been avoided, if only the disputants had perceived that in adjusting the sights different *focuses* may be made use of. One practitioner, for instance, will use the fine adjustment, taking a minute observation of the symptoms of the patient in great detail, and will find a simillimum to cover the picture. Another, working with a lower power, will take a more general view of the case, and select a medicine which he thinks corresponds to this. Both methods have given admirable results and both have their place in Homœopathy; and it is not at all my intention to dogmatize as to which is the better plan. I have succeeded with each one where the other has failed me."

Dr. Clarke does not, as before stated, narrow his use of the materia medica down to a very few remedies to be given over and over again by a sort of routine. On the contrary the whole boundless field of symptomatology is his for use. He makes this very clear in the following emphatic quotation: "It has been very truly said that any medicine may be required in any disease, and the case I have recorded in which *Crocus* played such a brilliant part is an illustration in point. If, therefore, I am asked, 'What medicines are good in cases of heart disease?' I must reply, 'All the medicines in the materia medica.' At the same time it is a very useful work to single out those medicines which have such characteristic action on the heart that they reproduce the features of the majority of the cases met with, and this I now propose to do." Then follows a list of medicines most used, with the indications for them.

